# Direct anterior and direct lateral approach in patients with femoral neck fractures receiving a total hip arthroplasty: a randomized controlled trial

**DOI:** 10.2340/17453674.2025.42847

**Published:** 2025-01-13

**Authors:** John Magne HOSETH, Tommy Frøseth AAE, Øystein Bjerkestrand LIAN, Tor Åge MYKLEBUST, Otto Schnell HUSBY

**Affiliations:** 1 Department of Orthopaedic Surgery, Health Møre and Romsdal HF, Kristiansund Hospital, Kristiansund; 2 Faculty of Medicine and Health Sciences, NTNU, Trondheim; 3 Department of Neuromedicine and Movement Science, NTNU, Trondheim; 4 The Clinical Research Unit, Health Møre and Romsdal HF, Ålesund; 5 The Cancer Registry of Norway, Oslo, Norway

## Abstract

**Background and purpose:**

The optimal approach to the hip joint in patients with displaced femoral neck fractures (dFNF) receiving a total hip arthroplasty (THA) remains controversial. We compared the direct lateral approach (DLA) with the direct anterior approach (DAA) primarily on Timed Up and Go (TUG), and secondarily on the Forgotten Joint Score (FJS), the Oxford Hip Score (OHS), EQ5D-5L, and the EQ5D-VAS.

**Methods:**

Between 2018 and 2023, we conducted a randomized controlled trial including elderly patients with dFNFs treated with THA. The primary outcome was the difference in TUG at 6 weeks postoperatively. Key secondary outcomes were TUG at 2, 12, and at 52 weeks postoperatively, and FJS, OHS, EQ5D-5L, and EQ5D-VAS at 2, 6, 12, and at 52 weeks postoperatively.

**Results:**

130 patients with a mean age of 78.6 (standard deviation 1.2) were allocated to DAA (n = 64) or DLA (n = 66). There was no statistically significant difference in TUG times at 6 weeks postoperatively between the DAA and the DLA, 16.0 s (95% confidence interval [CI] 13.2–18.7) vs 17.8 s (CI 15.1–20.4), estimated mean difference –1.8 s (CI –5.7 to 2.0). However, patients who underwent DAA had a significantly higher FJS at 2, 6, and 12 weeks.

**Conclusion:**

Among elderly patients with dFNF we found no difference between DAA or DLA regarding crude mobility as demonstrated with the TUG test, but patients treated with DAA showed better outcomes in the FJS in the early post-fracture period though not at 52 weeks.

The optimal surgical access to the hip joint in patients with femoral neck fractures (FNF) has been debated for decades [1]. The direct lateral approach (DLA) or Hardinge approach is easy to learn and gives low dislocation rates in the older FNF population [2]. The disadvantage of the DLA is that failure of gluteus medius can occur, which may lead to Trendelenburg gait with varying degrees of limping and discomfort [3]. The direct anterior approach (DAA) utilizes an intermuscular and an interneural interval, avoiding detachment of any muscles that could give rise to joint instability and a pathological gait pattern [4]. However, the DAA is associated with a learning curve and perioperative complications such as fractures and early femoral stem loosening [5]. Several studies have compared the DAA with the DLA in patients receiving a total hip arthroplasty (THA) due to hip osteoarthritis (OA). The general conclusion is that both groups perform equally well in terms of function, but that the DAA group may have a more rapid rehabilitation immediately after surgery, but not after 3 months [6-8]. FNF patients are usually older than patients receiving a THA due to hip OA, and they often have serious comorbidities [9]. Swift postoperative mobilization and rehabilitation is essential to reduce morbidity and mortality in the FNF population [10], and the DAA may therefore prove advantageous in the crucial immediate postoperative period. Few studies have compared the DAA with the DLA in elderly patients with FNF treated with a THA. In this study we aimed to investigate whether the DAA has superior outcomes to the DLA in elderly patients with FNF treated with a THA.

## Methods

### Trial design

A prospective randomized controlled trial was conducted in Kristiansund Hospital, Norway from November 2018 to February 2023, comparing the DAA with the DLA in patients with displaced FNF (dFNF) receiving a THA [11] based on the functional outcomes. The study is in accordance with the CONSORT guidelines.

### Participants

Patients above 50 years with a dFNF Garden type 3 or 4 were considered for inclusion if they were able to give written informed consent and were ambulatory before sustaining the fracture. The exclusion criteria were infection around the hip, alcohol use disorder, pathological fracture, bedridden patients, multitrauma patients, patients with life expectancy below 6 months (determined by consulting an internal medicine physician or an oncologist in case of advanced cancer disease), and patients with a known diagnosis of dementia or other causes of cognitive impairment. The presence or absence of exclusion criteria was determined through prior medical records, anamnesis, physical examination, and interview with next of kin. Exclusion criteria were set to minimize the loss to follow-up.

### Intervention

The DAA group was compared with the DLA group in a 1:1 ratio. All patients were treated with a THA.

### Outcomes

The primary outcome was to compare the Timed Up and Go (TUG) at 6 weeks postoperatively. TUG at 6 weeks was chosen because it measures crude mobilization at an early point in the rehabilitation [12]. Secondary outcomes were differences in the TUG at 2, 12, and 52 weeks postoperatively. Further secondary outcomes were differences in patient-reported outcome measures (PROMs), the Forgotten Joint Score (FJS), Oxford Hip Score (OHS), EQ5D-5L, and EQ5D-VAS postoperatively at 2, 6, 12, and 52 weeks. As the main focus of this article is the functional outcomes, the radiological results will be discussed in a separate article.

### Sample size

Given the heterogeneity in FNF patients we did sample size calculations for TUG, FJS, and the OHS to ensure that the number of patients included would give sufficient power to find a significant difference. The sample size calculated for TUG is based on 2 previous studies [12,13]. The minimal clinically important difference (MCID) was set to 3.4 s, and the standard deviation (SD) at 6.9. With a power of 80%, and a level of significance of 5%, 52 patients were required for each group. For the FJS we get 36 patients in each group when we set the MCID to 10 and the SD to 15 [14]. For the OHS we get 52 patients in each group when we set the MCID to 5 points and the SD to 9 [15]. Due to the high 1-year mortality in this study population, we estimated a 20% loss to follow-up (8). Consequently, the sample size is 65 patients in each group, giving a total of 130 patients.

### Randomization and stratification

Sequence generation and allocation. Stratification was performed to ensure equality among the groups. Stratification was based on 3 prognostic factors: (i) pre-fracture place of residence (i.e., home or residential care); (ii) pre-fracture functional status (i.e., using a walking aid or walking independently); (iii) American Society of Anesthesiologists (ASA) Class (i.e., Class I/II or III/IV/V). Further, patients were assigned to either DAA or DLA using a web-based randomization program [16]. In addition to randomization, this program stores the data prior to analysis.

### Implementation

The patients were included by the orthopedic resident on duty, while the principal investigator generated the allocation sequence and assigned patients to type of intervention.

### Blinding

There was no blinding of patients, surgeon, or physiotherapist.

### Surgery

2 experienced hip surgeons, both considered to be beyond the learning curve (> 100 procedures) for the DAA [17], performed all surgeries. The surgical procedure has already been described in detail in a recent study reporting exploratory results from the same study population [11].

### Primary outcome

TUG is a validated and reliable mobility assessment tool and measures the time that a person takes to rise from a chair, walk 3 m, turn around, walk back to the chair, and sit down [18]. TUG has good validity, responsiveness, and clinical utility when applied as a discharge measure in patients hospitalized for hip fractures, because it measures the ability to ambulate independently [19]. We standardized the walking aid to a rollator [20]. Patients first performed a test round, followed by 2 rounds where the average time was calculated.

### Secondary outcomes

The FJS measures the patient’s ability to forget about the joint replacement in everyday life. The FJS consists of 12 questions that are each answered on a 5-level scale, converted to a score that ranges from 0 (worst condition) to 100 points (best condition) [21]. The FJS has been validated in the hip joint fracture population [22]. The OHS is a validated questionnaire for patients undergoing THA for hip OA and FNF [23]. It consists of 12 items related to daily tasks directly influenced by poor hip function. The generic EQ5D-5L is a validated quality of life questionnaire consisting of 5 questions related to daily activities scored on a 5-point ordinal score scale [24]. As there is no Norwegian index, the EQ5D-5L was converted into a Swedish index score ranging from –0.314 (worst) to 1 (best). In EQ5D-VAS, respondents report their perceived health status with a grade ranging from 0 (worst possible health status) to 100 (best possible health status) [25].

The TUG and PROMs were administered by physiotherapists. In cases where transportation back and forth to the hospital for assessment was deemed too strenuous for patients, the study physiotherapists trained external physiotherapists in nursing homes and rehabilitation facilities in the study protocol to enable them to perform the testing.

### Statistics

Demographics and clinical parameters were analyzed as descriptive statistics using mean and SD. To analyze differences in TUG, FJS, OHS, EQ5D-5L, and EQ5D-VAS over time, we estimated repeated measures mixed-effect models (RMMEM) with random intercepts for each patient to facilitate the longitudinal structure of the data. Separate models for each of the 5 outcomes were estimated, and all models included study group, time, and the interaction between study group and time as covariates. From the estimated models, we predicted marginal means, and the corresponding 95% confidence intervals (CI), for each combination of time and study group. Pairwise differences were assessed using the Wald test. All analyses were performed using IBM SPSS version 29 (IBM Corp, Armonk, NY, USA) and STATA version 18.0 (StataCorp, College Station, TX, USA).

### Ethics, registration, data sharing, funding, use of AI, and disclosures

This randomized controlled trial obtained ethical approval from the Regional Ethics Committee in Norway (ID 2018/935). Written informed consent was obtained. The study was registered in clinicaltrials.gov with ID NCT03695497, Protocol ID 2018/935. Data sharing is possible upon request, which requires fulfillment of law regulations before distribution to foreign countries. No funding was required for this study. AI was not used. Complete disclosure of interest forms according to ICMJE are available on the article page, doi: 10.2340/17453674.2025.42847

## Results

**Figure 1:**
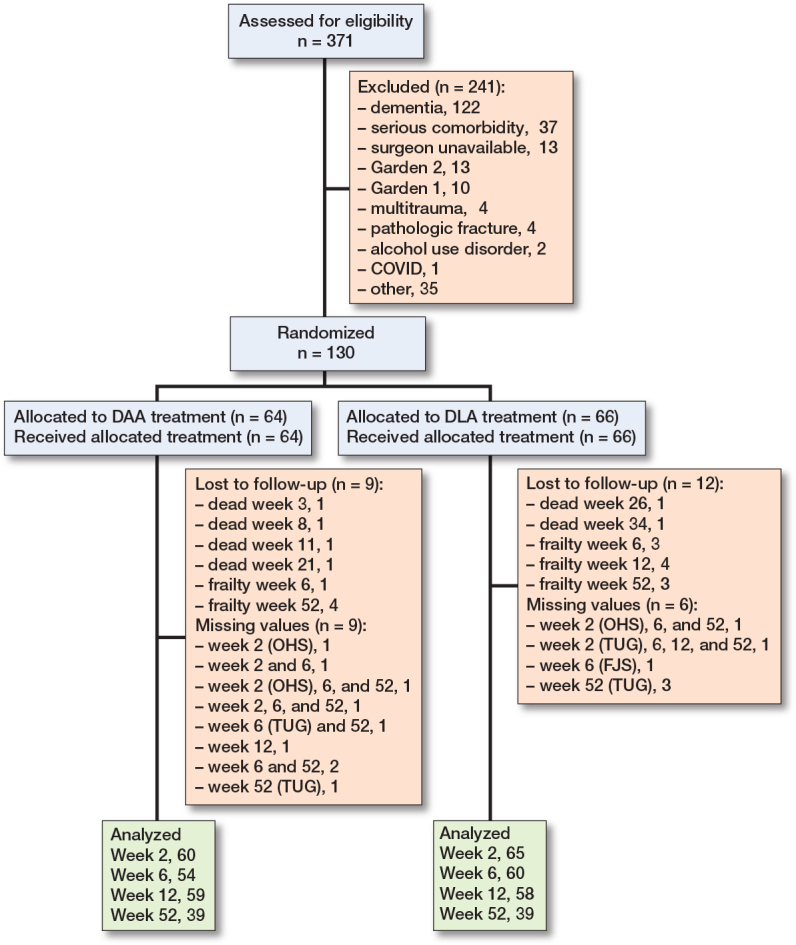
Consort flowchart.

Of 371 screened patients, 130 patients were included and 64 assigned to the DAA and 66 to the DLA group, respectively (Figure 1). 6 died in the study period of 52 weeks (4 from DAA and 2 from DLA). In most instances, the reason why some patients dropped out or missed certain check-ups were because they found the study controls too cumbersome. Baseline characteristics of the 2 groups were similar (Table 1). The hip-related complications were dislocation, periprosthetic fracture, infection, and Trendelenburg gait (see Table 3).

**Table 1:** Demographics and preoperative data of the study population

Factor	DAA	DLA
	n = 64	n = 66
Females, n (%)	42 (66)	41 (62)
Age (SD)	78.1 (1.2)	79.1 (1.2)
BMI (SD)	25.0 (0.6)	23.8 (0.5)
ASA grade		
1	0	2
2	17	16
3	43	46
4	4	2
Time to surgery in hours		
0–12	12	15
12–24	24	25
24–48	19	23
> 48	9	3
Pre-fracture walking aid	17	19
Admitted from residential care	2	3
General anesthesia, n (%)	8 (13)	9 (14)

DAA = direct anterior approach; DLA = direct lateral approach; BMI = body mass index; ASA = American Society of Anesthesiologists.

### Primary outcome

There was no statistically significant difference in TUG times at 6 weeks postoperatively between the DAA and the DLA, 16.0 s (CI 13.2–18.7) vs 17.8 s (CI 15.1–20.4), estimated mean difference –1.8 s (CI –5.7 to 2.0).

### Secondary outcomes

**Figure 2:**
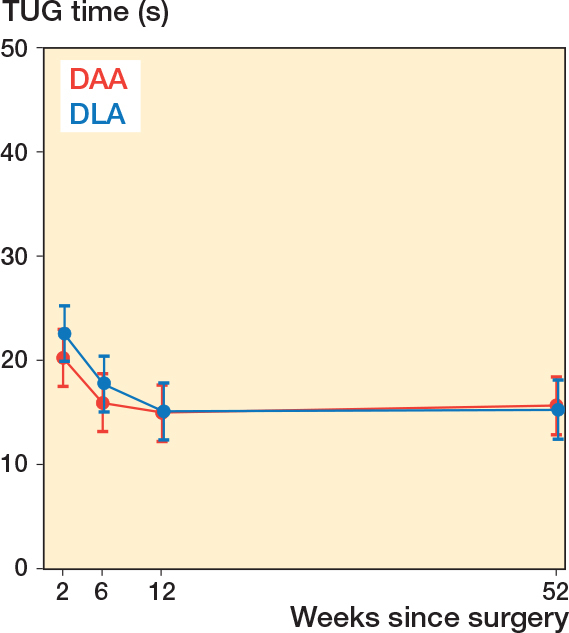
Least squares means for all time points by group for TUG, presenting 95% confidence interval for each group at different weeks following surgery.

There was no statistically significant difference in TUG times between the groups at 2, 12, and 52 weeks post-fracture (Table 2). TUG time was unchanged in both groups from 12 to 52 weeks postoperatively (Figure 2). The TUG times at 52 weeks for DAA and DLA were 15.6 s (CI 12.9–18.4) vs 15.2 s (CI 12.4–18.0), estimated mean difference 0.4 s (CI –3.5 to 4.4).

**Table 2:** Least squares means, differences, and number of completions in primary outcome (TUG), confirmatory secondary outcomes (FJS, OHS), and other secondary outcomes (EQ5D-5L, EQ5D-VAS)

Factor	DAA (CI)	n	DLA (CI)	n	Difference (CI)
TUG (s)					
2 weeks	20.2 (17.5–23.0)	62	22.6 (19.9–25.2)	65	–2.4 (–6.2 to 1.4)
6 weeks	16.0 (13.2–18.7)	54	17.8 (15.1–20.4)	61	–1.8 (–5.7 to 2.0)
12 weeks	14.9 (12.2–17.7)	59	15.1 (12.4–18.0)	58	–0.2 (–4.0 to 3.7)
52 weeks	15.6 (12.9–18.4)	39	15.2 (12.4–18.0)	39	0.4 (–3.5 to 4.4)
FJS					
2 weeks	52 (44–60)	62	39 (32–47)	66	13 (2 to 23)
6 weeks	62 (54–69)	55	48 (40–55)	60	14 (3 to 25)
12 weeks	70 (63–78)	59	55 (48–63)	58	15 (5 to 25)
52 weeks	76 (69–84)	40	66 (58–73)	42	10 (–0.1 to 21)
OHS					
2 weeks	29 (26–31)	60	26 (24–28)	65	3 (–0.2 to 6.0)
6 weeks	35 (33–38)	55	31 (29–33)	61	4 (1.2 to 7.3)
12 weeks	39 (37–41)	59	36 (33–38)	58	3 (0.1 to 6.2)
52 weeks	42 (40–44)	40	38 (36–40)	42	4 (0.4 to 7.0)
EQ5D-5L					
2 weeks	0.82 (0.78–0.87)	62	0.78 (0.74–0.83)	66	0.04 (–0.03 to 0.1)
6 weeks	0.85 (0.80–0.89)	55	0.86 (0.82–0.91)	61	–0.02 (–0.1 to 0.05)
12 weeks	0.87 (0.82–0.91)	59	0.87 (0.83–0.92)	58	–0.00 (–0.1 to 0.1)
52 weeks	0.91 (0.86–0.95)	40	0.85 (0.80–0.89)	42	0.06 (–0.01 to 0.1)
EQ5D-VAS					
2 weeks	57 (52–62)	62	62 (57–67)	66	–5 (–12 to 2)
6 weeks	64 (59–69)	55	65 (60–70)	61	–0.7 (–8 to 7)
12 weeks	67 (62–72)	59	68 (63–73)	58	–0.8 (–8 to 6)
52 weeks	69 (64–75)	40	68 (63–73)	42	1.0 (–6 to 9)

Baseline = 2 weeks after surgery. n = number of completed answers.

For abbreviations, see Table 1 and the following: CI = 95% confidence interval; TUG = Timed Up and Go; FJS = Forgotten Joint Score; OHS = Oxford Hip Score; VAS = visual analogue scale.

**Table 3:** Frequency of hip-related complications

Complication	Complications		Reoperations	
	DAA	DLA	DAA	DLA
Dislocation	2	1	1	0
Periprosthetic fracture	1	1	1	0
Infection	0	1	0	1
Trendelenburg gait	0	7	0	0

The FJS score was significantly higher for the DAA group at 2, 6, and 12 weeks postoperatively. The greatest difference was in week 12, 70 (CI 63–78) vs 55 (CI 48–63), estimated mean difference 15 (CI 5–25) (Table 2). The difference in week 6 was 62 (CI 54–69) vs 48 (CI 40–55), estimated mean difference 14 (CI 3–25). The difference at 52 weeks was 76 (CI 69–84) vs 66 (CI 58–73), estimated mean difference 10 (CI –0.1 to 21).

**Figure 3:**
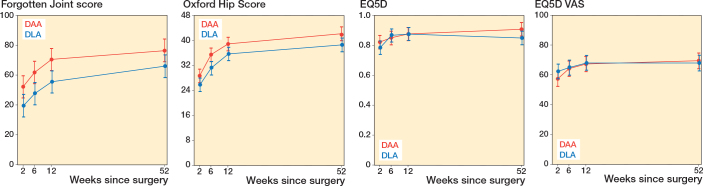
Least squares means for all time points by group for FJS, OHS, EQ5D-5L, and EQ5D-VAS, presenting 95% confidence interval for each group at different weeks following surgery.

The OHS score was higher for the DAA group at week 6, 35 (CI 33–38) vs 31 (CI 29–33), estimated mean difference 4 (CI 1.2–7.3) (Table 2). The difference at 52 weeks was 42 (CI 40–44) vs 38 (CI 36–40), estimated mean difference 4 (CI 0.4–7). There was no difference in the EQ5D-5L and EQ5D-VAS between the groups at the 4 follow-up points (Table 2, Figure 3).

Regarding destination of discharge, 40 (67%) patients were discharged to home in the DAA group, while this was the case for 33 (52%) patients in the DLA group.

## Discussion

We aimed to compare functional outcome and PROMs after THA in patients with dFNF treated either with the DAA or the DLA. The most important finding in this study was that the 2 groups had similar mean values in TUG time at 6 weeks post-fracture, and there was no clinical difference between the groups. In contrast, the FJS demonstrated a clinically significant difference in favor of the DAA group in the early post-fracture period, up to 3 months.

We also found a minor difference in the OHS between the groups at 6 weeks post-fracture in favor of the DAA, but the effect size was less pronounced than for the FJS.

In the literature the MCID for FJS is found to be 8 points [14,26]. In our study the FJS difference is above the MCID at every timepoint, reflecting clinical significance. However, the MCID for OHS is set at 5 points [15], rendering our results not clinically significant as the greatest OHS difference in our study was 4 points. Although a difference between the DAA and DLA was detected for OHS at week 6, a correlation with EQ5D results could not be demonstrated. Although the EQ5D diverged between the 2 groups from 12 weeks, the difference did not reach statistical significance.

The TUG measures crude mobility, while the FJS focuses on awareness of the hip joint. Our results suggest that patients treated with the DLA may be more conscious of their hip joint than those with the DAA.

The reason for the discrepancy in TUG and PROMS could reflect a floor effect in TUG due to the initial difficulty of conducting the test in a post-fracture setting. Similar results were found in a study by Ugland et al., where they compared the DLA with the anterolateral approach (ALA) in a FNF population receiving a hemiprosthesis (HA) [27]. They could not demonstrate a difference in TUG at any time point post-fracture; however, they could demonstrate a high risk for a positive Trendelenburg test in the DLA group, similar to our findings (Table 3). Saxer et al. compared the DAA with the DLA in 190 patients with FNF receiving a HA [28]. They measured TUG at 3 weeks postoperatively. Corresponding to our results, they could not find a statistically significant difference in TUG between the groups, but they showed an advantage for DAA in a subgroup of patients with low pre-fracture functional independence.

Another study comparing the DLA with the ALA in patients with FNF receiving a THA found a clinically significant difference in TUG times at 3 months post-fracture in favor of ALA [29]. Further, they could also demonstrate a difference in OHS at 3 months post-fracture in favor of ALA, aligning with our results. Equivalent to our study, they also excluded patients with dementia.

In a study comparing DAA with ALA in patients with FNF receiving an HE, Bűcs et al. found a significantly better Harris Hip Score in the DAA group compared with the ALA group at 2 and 6 weeks post-fracture [30]. Another study by Langlois et al. compared the DAA with the posterior approach (PA) in a population of FNF patients treated with HA. They found better TUG times at 6 weeks postoperatively for the DAA than for the PA [31]. Further, the PA was associated with a higher dislocation rate compared with the DAA, 20% and 3%, respectively. This corresponds to our findings of a low dislocation rate in the DAA. The PA for FNF is associated with an increased dislocation rate in several high-quality studies, which renders it less suitable for the FNF population [32,33]. It appears that the DAA and the ALA could offer some advantages in the early post-fracture phase in the FNF population compared with the DLA and PA [27-33].

The relatively low 1-year mortality in our population of approximately 5% can be ascribed to the strict inclusion criteria, excluding patients with severe multimorbidity.

### Limitations

One limitation is the fact that we have no pre-fracture status of our outcome measures, which would have been a valuable input in the RMMEM analysis. Another limitation is that our population is a selected group of patients with dFNF. We excluded one-third of the patients assessed for eligibility due to dementia, which are the patients at highest risk of dislocation. We also excluded patients with multimorbidity who were not expected to live beyond 6 months after inclusion. Our cohort is not entirely representative of the actual FNF population. Further, the lack of blinding is a potential source of bias, particularly for subjective outcome measures like the FJS and OHS. Another limitation is the number of missing values in both groups, although the RMMEM to some extent compensates for missing data. A noteworthy strength of this study is the randomized controlled design with careful stratification of certain prognostic factors, resulting in similar preoperative and demographic values between the groups.

### Conclusion

We found no difference in functional outcome between the groups when TUG was used at different times postoperatively. There was a slight difference in favor of DAA in the OHS at 6 weeks post-fracture, but the difference did not reach clinical significance. Based on the FJS, the patients in the DAA group seemed to be less aware of their hip than those in the DLA group at the early time points after the fracture.

## Notes

JMH: original idea, interpretation of data, drafting and revision of manuscript. TFA: interpretation of data, revision of manuscript. OHU: interpretation of data, revision of manuscript. TÅM: statistical analysis, interpretation of data. ØBL: revision of manuscript.

Handling co-editors: Cecilia Rogmark and Robin Christensen

*Acta* thanks Inari Laaksonen and Olof Sköldenberg for help with peer review of this manuscript.
